# Smoking, obesity and reoperation after elective open mesh repair of umbilical hernia: nationwide cohort study

**DOI:** 10.1093/bjsopen/zrag136

**Published:** 2026-10-13

**Authors:** Jeppe Fredberg, Erling Oma, Frederik Helgstrand, Lars N Jorgensen

**Affiliations:** Digestive Disease Centre, Bispebjerg Hospital, University of Copenhagen, Copenhagen, Denmark; Digestive Disease Centre, Bispebjerg Hospital, University of Copenhagen, Copenhagen, Denmark; Department of Surgery, Zealand University Hospital, Koege, Denmark; Department of Clinical Medicine, University of Copenhagen, Copenhagen, Denmark; Digestive Disease Centre, Bispebjerg Hospital, University of Copenhagen, Copenhagen, Denmark; Department of Clinical Medicine, University of Copenhagen, Copenhagen, Denmark

**Keywords:** body mass index, tobacco, hernia repair, elective surgery, postoperative complications

## Abstract

**Introduction:**

Evidence of the effects of obesity and smoking on complication-related reintervention after elective umbilical hernia mesh repair is limited. This study evaluated the association between these factors and reoperation after mesh repair.

**Methods:**

A nationwide cohort of elective open umbilical hernia repairs from 2017 to 2020 was identified from prospective registration in the Danish Hernia Database. Patients were followed until 31 December 2023 through nationwide registries and complete scrutiny of operative notes associated with succeeding reinterventions. Study outcomes were reoperation for any complication, recurrence, or non-recurrence complication with special reference to the repair method. Multivariable analyses included cause-specific hazard models with adjustment for body mass index (BMI), smoking status, sex, age, and hernia size.

**Results:**

Of the 2391 patients included in the study, 18.4% were smokers and 34.0% were obese (BMI > 30 kg/m^2^). The follow-up rate was 99.9% over a median of 4.65 years. Reoperation for any complication was increased in smokers compared with non-smokers (hazard ratio (HR) 1.68; 95% confidence interval (c.i.) 1.05 to 2.69; *P* = 0.032). Reoperation for non-recurrence complications was more frequent in smokers (HR 2.01; 95% c.i. 1.19 to 3.38; *P* = 0.009) and obese patients (HR 1.83; 95% c.i. 1.12 to 3.00; *P* = 0.016). Comparable risks of reoperation for hernia recurrence were found between smokers and non-smokers (HR 0.80; 95% c.i. 0.23 to 2.73; *P* = 0.721) and between patients with and without obesity (HR 0.73; 95% c.i. 0.26 to 2.09; *P* = 0.559).

**Conclusion:**

Reoperation for any complication after open umbilical hernia mesh repair was more frequent in smokers, whereas no independent association was observed between patients with and without obesity. Smoking and obesity were associated with an increased risk of reoperation for non-recurrence complications, while not affecting reoperation for hernia recurrence.

## Teaser

Although open elective umbilical hernia repair is common, controversy persists over surgical strategies for patients with preoperative risk factors. This nationwide study, using prospectively recorded perioperative data and complete long-term follow-up, including access to all reoperation notes, examined the impacts of smoking and obesity (body mass index > 30 kg/m^2^) on reoperation for complications in patients undergoing open mesh repair of umbilical hernia. The 5-year cumulative reoperation incidence was 5–6%. The efficacy of mesh repair in preventing reoperation for hernia recurrence was not associated with smoking status or body mass index. However, the benefit of mesh repair was offset by heightened early reoperation risk for wound-related complications in smokers and obese patients, leading to increased reoperation for any complication in smokers.

## Introduction

The prevalence of umbilical hernias may be as high as 25% among adults^1^. These defects in the linea alba at the umbilical ring account for approximately 50% of all ventral hernia repairs in Denmark^2^. With over 166 000 umbilical hernia repairs performed annually in the USA, this procedure ranks among the most frequently conducted operations in general surgery^3,4^.

Mesh repair reduces hernia recurrence by reinforcing the weakened abdominal wall^5–8^. However, this benefit may be offset by an increased risk of postoperative complications^5,9^, resulting in patient distress and substantial burden to the healthcare system^10^. Despite this risk, a meta-analysis on the topic suggests that mesh is superior to suture for the repair of primary ventral hernias^5^.

Increasing attention has been directed towards preoperative optimization in elective surgery, aiming to reduce postoperative complications and shorten the postoperative length of hospital stay^11,12^. Because obesity and smoking have been consistently identified as significant and modifiable risk factors for poor outcomes, they are frequently targeted in prehabilitation programs^13–16^. Although these two risk factors are common, no nationwide studies adjusted for prospectively collected perioperative data have specifically investigated their long-term effects on reoperation due to hernia recurrence or complications in patients undergoing mesh repair. The aim of this nationwide study was to evaluate the impacts of smoking and obesity on the risk of overall reoperation, as well as reoperation for hernia recurrence or other complications, after elective open mesh repair of umbilical hernia.

## Methods

### Study design and setting

This was a retrospective nationwide cohort study on patients who underwent open elective primary umbilical hernia repair using prospectively registered data from the Danish ventral hernia database (DVHD) from 1 January 2017 to 31 December 2020. Patients were followed up until subsequent abdominal surgery, emigration, death, or 31 December 2023, whichever came first. The study was conducted according to the RECORD statement for observational database studies^17^ and was registered with ClinicalTrials.gov (NCT06783036).

### Patients and data source

All patients were identified through the DVHD, established in 2007, which provides nationwide, lifelong surveillance of adult patients (age > 18 years) undergoing ventral hernia repair in all public and private surgical departments in Denmark^18^. The DVHD prospectively includes detailed patient demographics, hernia characteristics, and surgical techniques^18,19^, capturing over 90% of all ventral hernia repairs in Denmark^2,20^. In addition, it is the only publicly funded registry of its kind mandating procedure registration by Danish surgeons^21^. Registration of body mass index (BMI) and smoking status at the time of surgery has been mandatory since 2017.

Detailed perioperative data on patients undergoing open elective umbilical hernia repair were obtained from the DVHD, including hernia type, surgical approach, fascial defect size, repair technique, mesh type, BMI, and smoking status at the time of surgery.

Data on admissions, procedures, diagnoses, and co-morbidities, including the Charlson co-morbidity index^22^ (0, none; 1, mild; 2, moderate; ≥ 3, severe) were retrieved from the Danish National Patient Register (DNPR), which provides nationwide coverage of all hospital contacts^23^. Demographics and vital status were obtained from the Danish Civil Registration System (DCPR)^24,25^.

Patient data were merged across the DVHD, DNPR, and DCPR using patients’ unique Danish personal identification numbers, enabling complete follow-up and identification of all subsequent surgical procedures following the index hernia repair. Further details on the classification of subsequent operations, diagnoses, procedures, and linkage of databases are provided in *Fig. S1* and *Tables S1* and *S2*.

All medical records, including access to operative notes from public and private hospitals, were reviewed by two investigators (J.F., E.O.) to assess the relevance of subsequent operations to the index repair, documenting surgical indication, intraoperative findings, and procedure type. Any disagreement was resolved with a third specialist (L.N.J. or F.H.). Reviewers were not blinded to the index procedure.

Follow-up was discontinued after intra-abdominal surgery or procedures in proximity of the umbilicus to exclude uncertainty as to whether a later postoperative complication could be attributed to the index hernia repair or the subsequent surgical procedure.

### Inclusion criteria

The study included all patients with available data on BMI and tobacco use who underwent elective mesh repair for a primary umbilical hernia under general anaesthesia via an open approach as recorded in the DVHD. For patients undergoing multiple umbilical hernia repairs, the first (primary) repair recorded in the DVHD during the inclusion period was designated as the index repair.

### Exclusion criteria

Patients were excluded if the index surgery included inlay mesh placement, a minimally invasive approach, or an operation for hernia recurrence. Patients with hernia defects ≥ 10 cm were also excluded because the repair of defects of this size differs significantly in terms of surgical technique and complexity compared with the repair of smaller hernias.

### Outcomes

The primary outcome was reoperation for any complication during follow-up, comparing mesh repair across four subgroups defined by smoking status and the presence or absence of obesity (BMI > 30 kg/m^2^). The outcome of reoperation for any complication was a pooled outcome combining reoperations for recurrence and non-recurrence complications, thereby providing an estimate of the overall burden of reinterventions. To evaluate the individual contribution of these components, sensitivity analyses were performed separately for reoperations due to recurrence and non-recurrence complications. Non-recurrence complications were surgical site occurrences, including surgical site infections requiring general anaesthesia, as well as major complications such as bowel obstruction, intra-abdominal bleeding, and chronic mesh infection.

### Statistical analysis

Baseline characteristics are presented as counts with percentages for categorical data and as the median with interquartile range (i.q.r.) for continuous data, because all variables had a skewed distributed.

Clinically relevant risk factors were analysed in univariable analyses to identify independent predictors of hernia recurrence and other complications. Key risk factors were hernia size (maximal diameter), age, sex, BMI (for analysis of smoking status), and tobacco use (for analysis of BMI), and these were included in a multivariable cause-specific Cox proportional hazard regression analysis to adjust for competing risks. Results are reported as hazard ratios (HRs) with their 95% confidence interval (c.i.).

Cumulative incidence curves for reoperation for any complication, reoperation due to recurrence, and non-recurrence complications were generated using a competing risk analysis across groups stratified according to obesity and tobacco use. Gray’s test^26^ was used to compare cumulative incidence curves and compute 5-year cumulative incidence estimates.

Statistical comparisons in tables of patient demographics and reoperation characteristics used the Mann–Whitney rank-sum test for two independent groups, the Kruskal–Wallis rank-sum test for three or more independent groups, and Fisher’s exact test or Pearson’s χ^2^ test for associations between categorical variables in small and large samples, respectively.

Statistical analyses were performed using R, version 4.3.1 (R Foundation for Statistical Computing, Vienna, Austria) including CRAN and Bioconductor repositories with R studio version 2022.12.0. The following packages were used: tidyverse, ggplot2, survival, survminer, ggsurvfit, tidycmprsk, gtsummary, cowplot, and riskRegression.

### Ethical considerations

This study was approved by the Danish Health Data Authority (P-2023-193), the Center of Health (Journal number: R-23045342), the Regional Ethical Committees (J-23045663), and the Center for Research Support, Region Sjaelland (DOK-id: 496588). According to Danish law, individual patient consent was not required.

## Results

In all, 2440 eligible patients were identified in the DVHD (*Fig. 1*), of whom 2391 (98.0%) (median age 53.0 (i.q.r. 43.2–63.3) years) were included in the study. In this cohort, 441 patients (18.4%) were smokers, and 814 (34.0%) were obese. The median follow-up was 4.65 (i.q.r. 3.81–5.53) years. Additional patient, mesh, and reoperation characteristics are presented in *Table 1* and *Tables S3* and *S4*.

**Figure 1:**
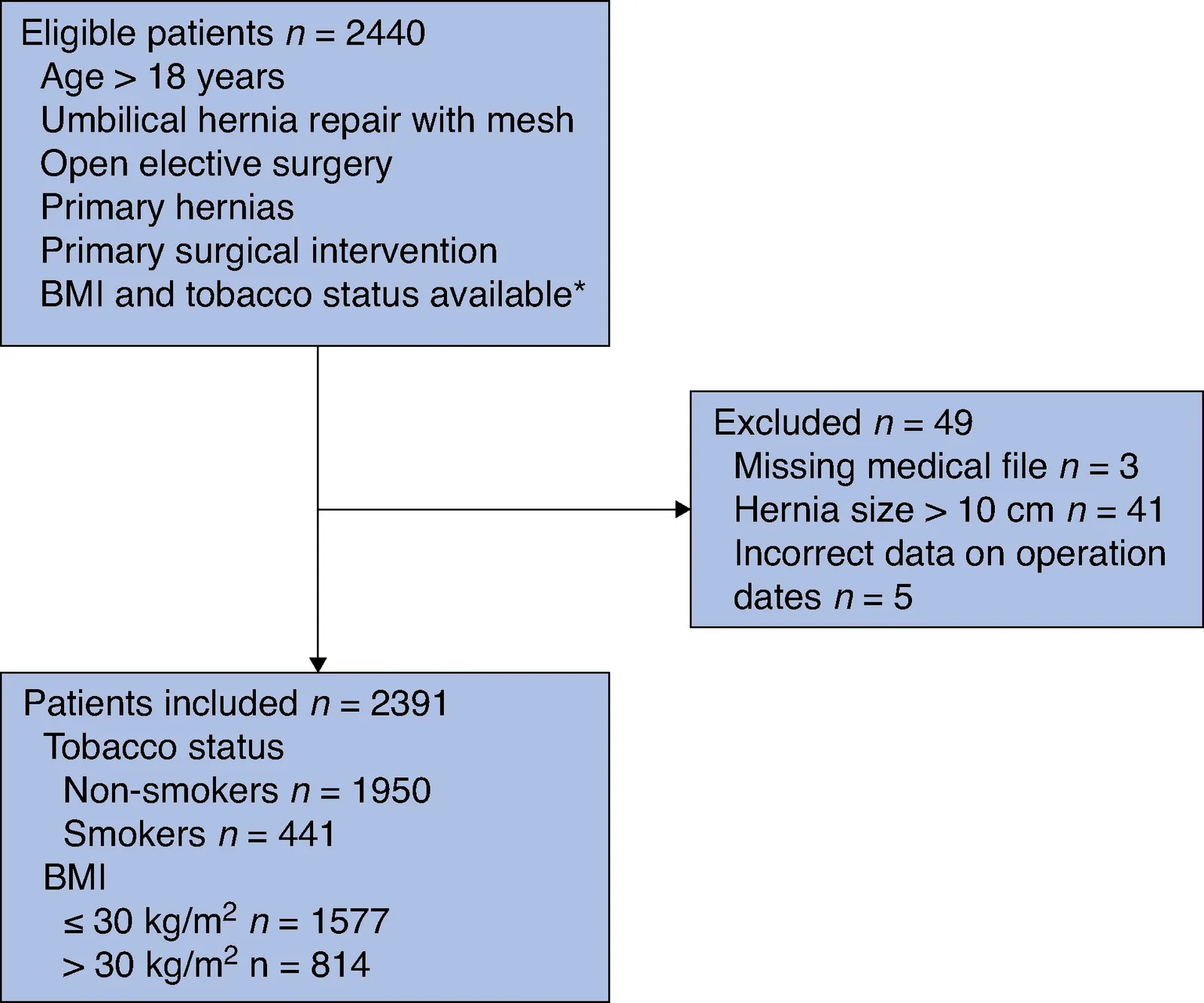
Study flow chart *Reporting of BMI and smoking status became mandatory in the Danish ventral hernia database on 1 July 2017. BMI, body mass index.

**Table 1:** Demographic characteristics of patients undergoing elective umbilical hernia mesh repair (*n* = 2391)

	Non-smoker (*n* = 1950)	Smoker (*n* = 441)	P*	BMI ≤ 30 kg/m^2^ (*n* = 1577)	BMI > 30 kg/m^2^ (*n* = 814)	P*
**Sex**			0.048			< 0.001
Female	480 (24.6%)	89 (20.2%)		432 (27.4%)	137 (16.8%)	
Male	1470 (75.4%)	352 (79.8%)		1145 (72.6%)	677 (83.2%)	
**Age (years)**			0.009			< 0.001
18–50	806 (41.3%)	198 (44.9%)		705 (44.7%)	299 (36.7%)	
> 50–60	489 (25.1%)	131 (29.7%)		367 (23.3%)	253 (31.1%)	
> 60–70	399 (20.5%)	70 (15.9%)		305 (19.3%)	164 (20.1%)	
> 70	256 (13.1%)	42 (9.5%)		200 (12.7%)	98 (12.0%)	
**BMI (kg/m^2^)**			0.010			
≤ 30	1301 (66.7%)	276 (62.6%)				
> 30	649 (33.3%)	165 (37.4%)				
**Smoking status**						0.010
Non-smoker				1301 (82.5%)	649 (79.7%)	
Smoker				276 (17.5%)	165 (20.3%)	
**Charlson co.morbidity index**			0.083			< 0.001
0	1403 (71.9%)	293 (66.4%)		1159 (73.5%)	537 (66.0%)	
1	277 (14.2%)	77 (17.5%)		215 (13.6%)	139 (17.1%)	
2	160 (8.2%)	37 (8.4%)		122 (7.7%)	75 (9.2%)	
≥ 3	110 (5.6%)	34 (7.7%)		81 (5.1%)	63 (7.7%)	
**Hernia defect size (cm)**			0.870			< 0.001
0–1	738 (37.8%)	161 (36.5%)		688 (43.6%)	211 (25.9%)	
> 1–2	914 (46.9%)	212 (48.1%)		715 (45.3%)	411 (50.5%)	
> 2	298 (15.3%)	68 (15.4%)		174 (11.0%)	192 (23.6%)	

Values are *n* (%). BMI, body mass index. *Pearsons chi-squared test.

### Smoking

The 5-year cumulative incidence of reoperation for any complication related to the index hernia repair was 5.5% (24) and 3.4% (64) following mesh repair among smokers and non-smokers, respectively (*P* = 0.028). A relatively high proportion of reoperations occurred within 30 days after mesh repair in both the smoker and non-smoker groups (*Fig. 2*). In multivariable analysis, smoking was associated with a 68% increased risk of reoperation for any complication compared with non-smoking (*P* = 0.032; *Table 2*). In addition, smoking and increasing hernia size were significantly associated with reoperation for any complication (*P* = 0.032 and *P* = 0.024, respectively).

**Figure 2:**
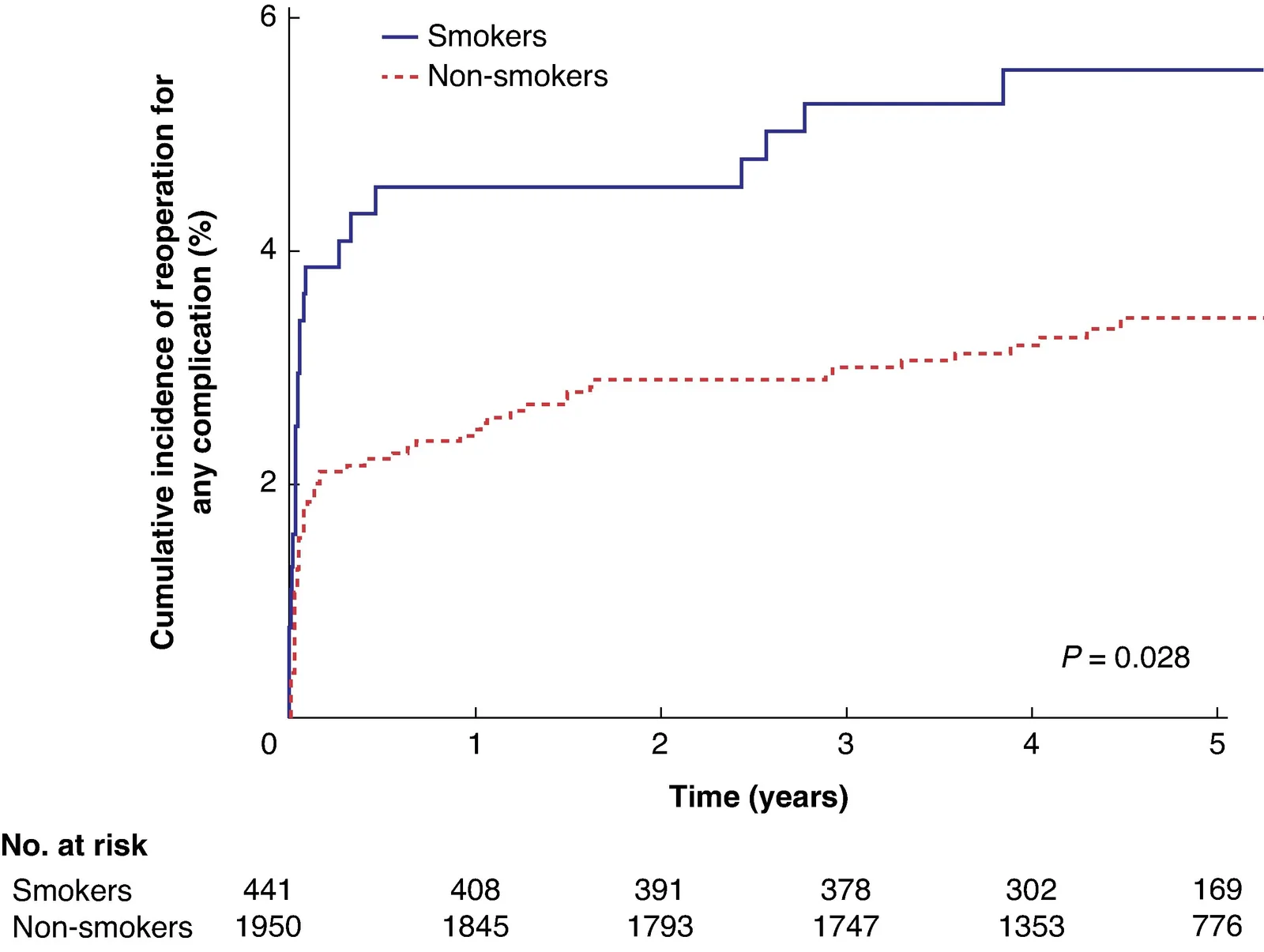
Reoperation for any complication following elective open mesh repair of umbilical hernia in smokers *versus* non-smokers

**Table 2:** Multivariable cause-specific Cox proportional hazard regression analysis of factors associated with reoperation by indication after elective umbilical hernia mesh repair

	Any complication		Hernia recurrence		Non-recurrence complications	
	Hazard ratio	*P*	Hazard ratio	*P*	Hazard ratio	*P*
**Sex**						
Female	1.00		1.00		1.00	
Male	0.70 (0.44, 1.13)	0.145	0.35 (0.14, 0.88)	0.025	0.93 (0.52, 1.67)	0.816
**Age (per year)**	0.99 (0.97, 1.00)	0.149	0.98 (0.95, 1.01)	0.220	1.00 (0.98, 1.01)	0.375
**Smoking status**						
Non-smoker	1.00		1.00		1.00	
Smoker	1.68 (1.05, 2.69)	0.032	0.80 (0.23, 2.73)	0.721	2.01 (1.19, 3.38)	0.009
**BMI (kg/m^2^)**						
≤ 30	1.00		1.00		1.00	
> 30	1.50 (0.97, 2.33)	0.066	0.73 (0.26, 2.09)	0.559	1.83 (1.12, 3.00)	0.016
**Hernia size (per cm)**	1.22 (1.03, 1.45)	0.024	1.14 (0.76, 1.70)	0.521	1.25 (1.03, 1.52)	0.024

Values in parentheses are 95% confidence intervals. BMI, body mass index.

The 5-year cumulative incidence of reoperation for hernia recurrence was comparable among smokers and non-smokers (0.7% (3) and 1.0% (18), respectively; *P* = 0.620 *Fig. S2a*). In contrast, reoperation for non-recurrence complications was more frequent among smokers than non-smokers (4.8% (21) *versus* 2.4% (46), respectively; *P* = 0.006; *Fig. S2b*). In multivariable analysis, smokers and non-smokers were at comparable risk of reoperation for hernia recurrence (HR 0.80; *P* = 0.721), whereas smoking was linked to a higher risk of reoperation for non-recurrence complications (HR 2.01; *P* = 0.009; *Table 2*).

### Obesity

Mesh repair among obese and non-obese patients was associated with 5-year cumulative incidences of reoperation for any complication related to the index hernia of 4.9% (39) and 3.2% (49), respectively (*P* = 0.037; *Fig. 3*). In multivariable analysis, patients with and without obesity and had a comparable risk of reoperation for any complication (*P* = 0.066; *Table 2*). Increasing hernia size and smoking significantly predicted reoperation for any complication (*P* = 0.024 and *P* = 0.032, respectively).

**Figure 3:**
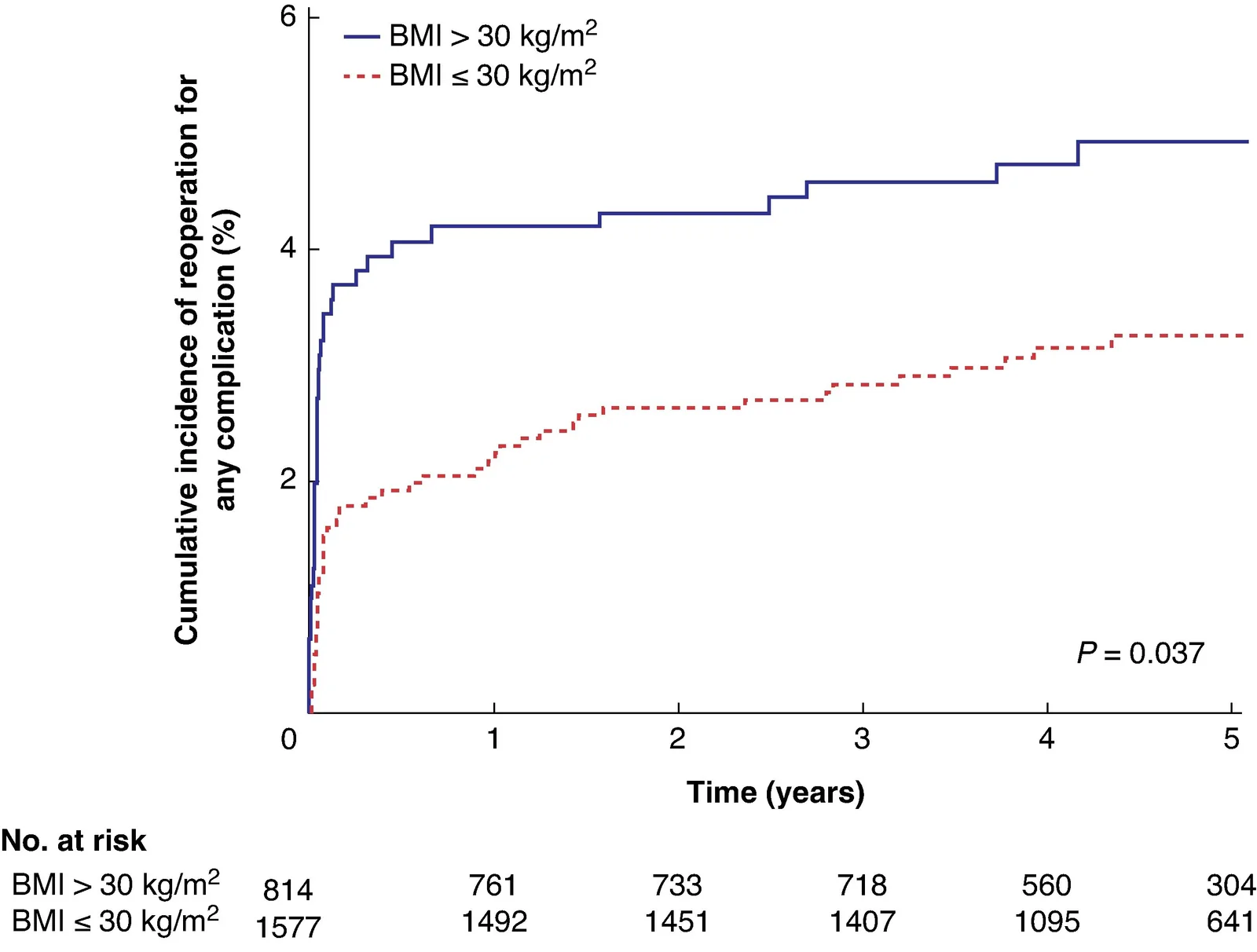
Reoperation for any complication following elective open mesh repair of umbilical hernia in patients with (BMI > 30 kg/m^2^) *versus* without (BMI ≤ 30 kg/m^2^) obesity. BMI, body mass index.

Addressing reoperation for hernia recurrence, the 5-year risks were 0.7% (5) *versus* 1.1% (16) among patients with and without obesity, respectively (*P* = 0.320; *Fig. S3a*). Conversely, reoperation for non-recurrence complications was more frequent after mesh repair in patients with than without obesity (4.2% (34) *versus* 2.1% (33); *P* = 0.003; *Fig. S3b*). In multivariable analysis, the risk of reoperation for hernia recurrence was comparable between patients with and without obesity (*P* = 0.559). Male sex was significantly associated with a lower risk of reoperation for hernia recurrence (*P* = 0.025). In addition, independent predictive risk factors for reoperation for non-recurrence complications were obesity (HR 1.83; *P* = 0.016) and hernia size (1.25; *P* = 0.024; *Table 2*).

## Discussion

This nationwide study comprehensively examined the effects of smoking and obesity on reoperation for complications after open mesh repair for umbilical hernia, with complete long-term follow-up and detailed operative note review. The 5-year cumulative incidence of reoperation was only 5–6%, and thus substantially less than after incisional hernia repair^27^. Mesh repair was associated with increased reoperation for any complication in smokers. Neither smoking nor obesity significantly influenced the rate of reoperation for hernia recurrence. The benefit of mesh repair was offset by an increased early risk of reoperation for mostly wound-related complications in smokers and patients with obesity.

A recent systematic review and meta-analysis of 25 cohort studies on primary ventral or incisional hernia repair, including 69 295 patients (20.5% smokers), also found an association between smoking and wound morbidity, but not reoperation^28^. Other studies similarly identify smoking as a risk factor for wound complications^29–31^. The present study, which relied on national tracking of all patients and comprehensive long-term follow-up, including scrutiny of all reoperation notes, strongly suggests that smoking also elevates the rate of reoperation for non-recurrence complications.

Howard *et al*.^32^ recently reported on the effects of smoking in a retrospective cohort of 12 233 patients who underwent elective open or laparoscopic ventral hernia repair and found no differences in 30-day complications or reoperation. That study^32^ assessed 30-day outcomes, whereas the nearly 5-year median follow-up in the present study, including exclusively open mesh repairs, showed that only 63% of non-recurrence reoperations occurred within 30 days, and operations for recurrence appeared gradually throughout the entire follow-up period. Because minimally invasive surgery is associated with fewer wound complications^33–35^, this methodological difference may partly explain the divergent findings. Other studies^36–38^ support the duration of follow-up being associated with hernia recurrence rates.

Obesity is a major focus within abdominal wall surgery. Currently, 43% of adults worldwide are categorized as overweight and 13% as obese^39^. In addition to increasing the incidence of primary ventral hernia^40^, a propensity score-matched analysis of 168 214 ventral hernia repairs found an increased risk of 30-day wound morbidity among obese patients, with risk rising progressively with BMI and already significantly elevated at BMI between 25 and 30 kg/m^241^. These studies support the findings of the present study of an almost twofold increase in reoperation for non-recurrence complications in obese patients undergoing open mesh repair. Parker *et al*.^15^, including 274 studies in a meta-analysis on predictors of ventral hernia recurrence, found that BMI > 30 kg/m^2^ significantly increased the risk of recurrence, contrasting with the present findings. The analysis of Parker *et al*.^15^ did not distinguish between suture and mesh repair and relied on univariate predictor analyses, which may partly explain the differing results. Another meta-analysis^42^, including five studies with 465 patients evaluating the effect of preoperative weight loss, reported no differences in hernia recurrence, surgical site infection, reoperation, or overall complications. This is consistent with the present findings on reoperation for any complication and recurrence, but not for non-recurrence complications. In contrast to the present cohort of exclusively open repairs, most patients in the meta-analysis underwent laparoscopic hernia repair, which has been associated with fewer wound complications^33–35^. Consistent with the literature, the present study suggests that postoperative wound morbidity is related to the combination of obesity and open mesh repair^43^.

This study only assessed complications diagnosed at the first subsequent reoperation, leaving purely clinically observed events and later reoperations uncounted. Furthermore, previous studies compared smokers to non-smokers and patients with obesity to non-obese patients, whereas the present study compared both these subgroups, providing more granular data on high-risk patients. As yet, no previous studies have specifically evaluated mesh efficacy in relation to both smoking and obesity.

The primary strength of the present study is its nationwide design using prospectively collected perioperative data from the DVHD, which allowed statistical adjustment for important demographic and technical variables. The DVHD captures over 90% of ventral hernia operations in Denmark across public and private hospitals, enhancing the external validity of the recorded data. By linking data from DNPR and DCPR, 99.9% long-term follow-up was obtained, including assessment of operative notes of all reinterventions, which allowed detailed evaluation of their relation to the index hernia repair. Contrary to many recent large-scale studies^14,16,32,41,44^, the present study specifically included patients undergoing elective open umbilical hernia repair with mesh. This is important because these hernias differ substantially from incisional hernias^45^.

Nevertheless, this study has limitations. The observational design introduced potential selection bias and residual confounding from baseline imbalances. Mesh type and positioning are potentially influenced by patient-specific clinical factors, such as age, hernia size, BMI, smoking status, and co-morbidities. This may have introduced selection bias and baseline imbalances that cannot be entirely adjusted for, even with multivariable analyses, thereby allowing residual confounding. A higher proportion of obese than non-obese patients presented with hernias > 2 cm , which may have influenced the choice of surgical technique, as indicated by larger mesh size in obese patients.

Mandatory registration of BMI and smoking in the DVHD began in 2017, limiting earlier data and resulting in only 441 smokers undergoing mesh repair, which may have reduced statistical power. This study assessed reoperation after umbilical hernia repair, thus only capturing a fraction of cases and underestimating the true incidence of complications, including hernia recurrence^46^. Similarly, only non-recurrence complications requiring reintervention under general anaesthesia were included, contributing to lower complication rates. Moreover, follow-up was restricted to the first intra-abdominal reoperation, regardless of whether it was directly related to the index hernia repair. This approach was chosen because of the difficulty in determining whether a later complication was attributable to the initial hernia repair or to subsequent, unrelated, intra-abdominal surgery. Consequently, reported outcomes are conservative. Patients undergoing unrelated operations after their index hernia repair may have experienced complications associated with the initial repair that were not captured.

In this study of patients undergoing elective open mesh repair of umbilical hernia, smoking was associated with an increased risk of reoperation for any complication, whereas both smoking and obesity were associated with a higher risk of reoperation for non-recurrence complications. The rationale of preoperative optimization, including smoking cessation and weight reduction, should be encouraged to prevent wound-related complications, bearing in mind that a potential absolute risk reduction of 2% represents only a modest benefit relative to the burden these interventions may pose.

## Supplementary Material

zrag136_Supplementary_Data

## Data Availability

The authors had complete access to the study data. According to Danish law, the current data from public health registries cannot be made accessible to other researchers.
