# Supplementary material for: Smoking, obesity and reoperation after elective open mesh repair of umbilical hernia: nationwide cohort study
Source: BJS Open. 2026 Oct 13;10(5):zrag136. doi: 10.1093/bjsopen/zrag136 (PMC13660690; doi:10.1093/bjsopen/zrag136)
Supplement: Supplementary file 1 [file zrag136_supplementary_data.docx]

**Title:** Smoking, obesity and reoperation after elective open mesh repair of umbilical hernia: Nationwide cohort study

Authors:

Jeppe Fredberg MD^1^

Erling Oma MD PhD^1^

Frederik Helgstrand MD DMSc^2,3^

Lars N. Jørgensen MD DMSc^1,3^

^1^Affiliation: Digestive Disease Centre, Bispebjerg Hospital, University of Copenhagen, Copenhagen, Denmark

^2^Affiliation: Department of Surgery, Zealand University Hospital, Koege, Denmark

^3^Affiliation: Department of Clinical Medicine, University of Copenhagen, Copenhagen, Denmark

**Corresponding author:** Lars Nannestad Jørgensen, Digestive Disease Centre K, Bispebjerg Hospital, Bispebjerg Bakke 23, DK-2400 Copenhagen NV, Denmark; **ORCID ID:** 0000-0001-7465-5374; **X:** @larsnjorgensen

| **Supplementary Methods** |  | | |
| --- | --- | --- | --- |
| Figure S1: Linkage of databases | | | *page 2* |
| Table S1: Categorisation of surgical codes  Table S2: Categorisation of relevant action diagnoses, add-on codes for procedure and diagnosis codes | | | *pages 3-4*  *page 5* |
| **Supplementary Tables and Figures** |  | | |
| Table S3: Intraoperative data, open elective mesh repair of umbilical hernia | | *page 6* | |
| Table S4: Pathology at reoperation, unadjusted rates  Figure S2: Reoperation for recurrence and non-recurrence complication after open elective mesh repair of umbilical hernia, smokers versus non-smokers  Figure S3: Reoperation for recurrence and non-recurrence complication after open elective mesh repair of umbilical hernia, obese (BMI > 30 kg/m^2^) versus non-obese (≤ 30 kg/m^2^) patients | | *page 7*  *page 8*  *page 9* | |
| **Supplementary References** | *page 10* | | |
|  |  | | |

**Supplementary Methods**

Figure S1: Linkage of databases

Alt text: Flow diagram showing linkage of databases and patient selection. Of 9 177 patients screened, 6 737 were excluded, leaving a study population of 2 440 patients for open umbilical hernia repair with mesh as the primary surgical procedure.

## Table S1: Categorisation of surgical codes^1^

| **Surgical specialty** | **Category** | **Surgical codes** |
| --- | --- | --- |
| Operations on endocrine organs (KB*) | Stop of follow-up, unrelated | KBCA30 |
| Operations on the heart and large intrathoracic blood vessels (KF*) | Continuous follow-up | KFCA50 |
|  | Stop of follow-up, possibly related. | KJAD10, KJAD11, KJAD30, KJAD96, KJAD97, KJAE10, KJAE11, KJAE14A, KJAE30, KJAF10, KJAF11, KJAF14A, KJAF30, KJAF97, KJAG00, KJAG01, KJAG60, KJAG96A, KJAH00, KJAH01, KJAH01A, KJAH30, KJAH33, KJAH96, KJAK04, KJAL06, KJAL10, KJAL11, KJAL21, KJAP00, KJAP01, KJAW96, KJBC01, KJCC11, KJDC96, KJDC97, KJDW96, KJFA10, KJFA70, KJFA81, KJFB00, KJFB01, KJFB20, KJFB21, KJFB30, KJFB30A, KJFB31, KJFB31A, KJFB41, KJFB43, KJFB44, KJFB46, KJFB47, KJFB51, KJFB60, KJFB61, KJFB97, KJFC00, KJFC01, KJFF01, KJFF11, KJFF27, KJFG40, KJFK00, KJFK01, KJFK10, KJFK97, KJFL10, KJFL11, KJFL96, KJWA00, KJWB00, KJWC00, KJWD00, KJWE00, KJWW96, KJAA00, KJAA00, KJAA00A, KJAA10, KJAA11, KJAA96, KJAA97, KJAA97, KJAC30, KJCA52B, KJFA22, KJLW98A, KJCA38, KJDA55, KJDH08, KJFA12, KJFA32, KJFA55B, KJGA75, KJHB96, KJJA10A, KJKA30, KJHD96 |
| Operations on the digestive organs and spleen (KJ*) | Stop of follow-up, unrelated | KJAB11, KJAC11, KJAK10, KJAK11, KJAL30, KJAL31, KJAW90, KJAW91, KJBB01, KJCC00, KJCE21, KJDA60, KJDA61, KJDB00, KJDB10, KJDB10A, KJDB10B, KJDC10, KJDE01, KJDF11, KJDF41, KJDH00, KJDH70, KJDW97, KJDW97A, KJEA00, KJEA01, KJFG00A, KJFG10, KJFG20, KJFG20A, KJFG26, KJFG41A, KJFH11, KJFM00, KJGB00, KJGB01, KJGB04, KJGB11, KJGB31, KJGB33, KJJA31, KJJA41, KJJB10, KJJB40, KJJB53, KJJC00, KJKA11, KJKA20, KJKA21, KJKB01, KJKB11, KJKB21, KJLC10, KJLC30, KJMA10, KJAA10, KJAA11 |
|  | Continuous follow-up | KJAB00, KJAB10, KJAB30, KJAB96, KJAC10, KJAC30, KJAL00, KJBC02, KJCA08, KJCA12, KJCA20, KJCA22, KJCA32, KJCA35, KJCA38, KJCA52B, KJCA55, KJCC10, KJCF00, KJCF12, KJDA05, KJDA08, KJDA22, KJDA32, KJDA35, KJDA42, KJDA55, KJDH05, KJDH08, KJDH15, KJDH18, KJDH25, KJDH28, KJDH32, KJFA12, KJFA15, KJFA22, KJFA32, KJFA38, KJFA42, KJFA45, KJFA48, KJFA52, KJFA55, KJFA55A, KJFA55B, KJFA68, KJGA05, KJGA52A, KJGA58A, KJGA73, KJGA75, KJHA00, KJHA00A, KJHA10, KJHA20, KJHA30, KJHB00, KJHB10, KJHB30, KJHB96, KJHB96A, KJHB96B, KJHD00, KJHD20, KJHD30, KJHD30A, KJHD42, KJHD96, KJHW96, KJJA10A, KJJA20, KJJA26, KJJA43, KJJA43A, KJJA43B, KJKA16, KJKA30, KJKB30, KJKB33, KJKE02, KJKE12, KJKE15, KJKE18, KJKE18A, KJKE22, KJKE32, KJKE98, KJLA20, KJLB28, KJLD12C, KJLT00, KJLW98A, KJMB00, KJWW98, KJAA00A |
| Obstetric surgeries (KM*) | Stop of follow-up, possibly related | KMBA03 |
|  | Stop of follow-up, unrelated | KMCA10B |
|  | Continuous follow-up | KMBA03 |
| Operations on the female genital organs (KL*) | Stop of follow-up-, possibly related | KLAE10, KLAE11, KLAF00, KLBD00, KLBE00, KLEF00, KLEF03, KLEF10, KLEF53B, KLEF60, KLEF96, KLEG10, KLFE20, KLAA10, KLAA10A, KLDC96, KLEF00B, KLEF40, KLEW96, KLFB10 |
|  | Stop of follow-up, unrelated | KLAC00, KLAC01, KLAC31, KLAD01A, KLAF01, KLAF10, KLAF11, KLAG21, KLBC21, KLBE01, KLBE03, KLBE11, KLCA24, KLCB10, KLCB11, KLCD00, KLCD01, KLCD01A, KLCD01B, KLCD30, KLDC13B, KLGA10, KLGA11, KLGA21, KLWA00, KLWW96 |
|  | Continuous follow-up | KLCA06, KLCA10, KLCA30, KLCA96, KLCB25, KLDC03, KLDC10, KLDC20, KLDC96, KLEF00, KLEF00B, KLEF03, KLEF10, KLEF40, KLEF53B, KLEF60, KLEF96, KLEG10, KLEW96, KLFB10, KLFE20, KLAA10, KLAA10A |
| Operations on peripheral blood vessels and the lymphatic system (KP*) | Stop of follow-up, possibly related | KPCQ10, KPCQ30, KPDU84, KPDH35, KPCP30 |
|  | Stop of follow-up, unrelated | KPDG10, KPDG20, KPDP30, KPDQ10, KPDQ30, KPDU83 |
|  | Continuous follow-up | KPCP30, KPCQ10, KPCQ30, KPDH35, KPDU84 |
| Operations on the skin and subcutaneous tissue (KQ*) | Stop of follow-up, possibly related | KQBA00, KQBA00A, KQBA10, KQBA30, KQBB00, KQBB00A, KQBB00B, KQBB05, KQBB10, KQBB99, KQBC00, KQBE00, KQBE10, KQBE10A, KQBE10R, KQBE30, KQBE99, KQBG20, KQBG30, KQWB00, KQXE99, KQAB05A, KQAC00, KQAE10, KQAE10A, KQAJ15, KQAJ30, KQAJ35, KQBA10B, KQBA10D, KQBA40, KQBA40A, KQBA50, KQBA50A, KQBA99, KQBE10B, KQBE10CC, KQBE20, KQBG00, KQBJ00, KQBJ05B, KQBJ05C, KQBJ30, KQBJ30A, KQBJ30B, KQCA99A, KQCB00, KQCB00A, KQCB05, KQCB10, KQCE10A, KQCE10B, KQCJ00, KQCJ05A, KQDA40, KQDB00, KQDB10, KQDB99, KQDE00, KQDE10, KQDJ00, KQBA10A, KQBE70, KQCA50, KQCA99, KQXJ00, KQAA40, KQBA60, KQBD00, KQBJ05, KQBJ05A, KQBJ99, KQDE10A, KQDJ05A |
|  | Continuous follow-up | KQAB05A, KQAC00, KQAE10, KQAE10A, KQAJ15, KQAJ30, KQAJ35, KQBA10A, KQBA10B, KQBA10D, KQBA40, KQBA40A, KQBA50, KQBA50A, KQBA60, KQBA99, KQBD00, KQBE10B, KQBE10CC, KQBE20, KQBE70, KQBG00, KQBJ00, KQBJ05, KQBJ05A, KQBJ05B, KQBJ05C, KQBJ30, KQBJ30A, KQBJ30B, KQBJ99, KQCA50, KQCA99, KQCA99A, KQCB00, KQCB00A, KQCB05, KQCB10, KQCE10A, KQCE10B, KQCJ00, KQCJ05A, KQDA40, KQDB00, KQDB10, KQDB99, KQDE00, KQDE10, KQDE10A, KQDJ00, KQDJ05A, KQXJ00, KQAA40 |
| Procedures performed during surgery (KW*) | Stop of follow-up, possibly related | KWAB01, KWAA01 |
| Operations on the urinary tract, male genital organs, and retroperitoneal tissue (KK*) | Stop of follow-up, possibly related | KKDH70, KKKA10, KKBV07, KKBV10, KKCD32, KKDC00, KKDD32, KKDW96 |
|  | Stop of follow-up, unrelated | KKAC00, KKAC01, KKAC21, KKAD01, KKAD11, KKAH41, KKAS10, KKAS20, KKAS70, KKBH20, KKBJ10, KKBV01, KKCC11, KKCD20, KKCH00, KKDH50, KKEC01, KKEC01A, KKEC01B, KKEC01C, KKKA20A, KKKB10 |
|  | Continuous follow-up | KKAE01, KKAE12, KKAJ00, KKBD22, KKBE12, KKBE32, KKBH51, KKBV00, KKBV02, KKBV02A, KKBV05, KKBV07, KKBV10, KKCD32, KKDC00, KKDD32, KKDH00, KKDH70, KKDV10, KKDV12, KKDW96, KKFA40, KKFC00, KKFD00, KKGA96, KKKA10, KKWD00, KKWE00, KKWW96, KKAA01 |

This table includes all surgical procedure codes describing the subsequent operations performed after the index hernia repair. Follow-up was discontinued after any intra-abdominal procedure because of the uncertainty regarding whether later complications could be attributed to the index hernia repair or the subsequent operation. In contrast, unrelated superficial procedures did not result in discontinuation of follow-up. Subsequent procedures were also classified according to whether they were considered potentially related to the index hernia repair. All possibly related medical files were subjected to a detailed review.

## Table S2: Categorisation of relevant primary diagnoses, supplementary procedure, and diagnosis codes^1^

| Relevant primary diagnoses | DK42, DK430, DK431, DK432, DK436, DK436a, DK436b, DK437, DK439, DK450, DK450f, DK451, DK451f, DK458, DK458f, DK46 |
| --- | --- |
| Relevant supplementary procedure codes | BNPA80, BNPA92, KJAH2, KJAP01, KJFB00, KJFK10, KWAB01 |
| Relevant diagnosis codes | DK42*, DK430, DK431, DK432, DK436, DK436a, DK436b, DK437, DK439, DK450, DK450f, DK451, DK451f, DK458, DK458f, DK46 |
| Relevant supplementary diagnosis codes | DK429, KJAD14A, KJAD30, KJAE, KJAF |

Patient records associated with relevant primary diagnosis codes, supplementary procedure codes, primary diagnosis codes, and supplementary diagnosis codes were subjected to detailed review.

**Supplementary Tables and Figures**

## Table S3: Intraoperative data, open elective mesh repair of umbilical hernia, n = 2 391

| **Characteristic** | **Non-Smoker N = 1 950** | **Smoker N = 441** | ***P* value^a^** |  | **BMI ≤ 30 kg/m^2^ N = 1 577** | **BMI > 30 kg/m^2^ N = 814** | ***P* value^a^** |
| --- | --- | --- | --- | --- | --- | --- | --- |
| Mesh type, n (%) |  |  | .230 |  |  |  | .570 |
| Polypropylene | 1 268 (65.1) | 274 (62.1) |  |  | 1 015 (64.4) | 527 (64.8) |  |
| Coated polypropylene | 475 (24.4) | 116 (26.3) |  |  | 416 (26.4) | 175 (21.5) |  |
| Coated polyester | 15 (0.8) | 4 (0.9) |  |  | 11 (0.7) | 8 (1.0) |  |
| Polyester | 165 (8.5) | 38 (8.6) |  |  | 113 (7.2) | 90 (11.1) |  |
| Polypropylene + ePTFE | 17 (0.9) | 7 (1.6) |  |  | 17 (1.1) | 7 (0.9) |  |
| Biosynthetic | 1 (<0.1) | 0 (0) |  |  | 1 (<0.1) | 0 (0) |  |
| Other | 7 (0.4) | 2 (0.5) |  |  | 3 (0.2) | 6 (0.7) |  |
| Unknown | 2 (0.1) | 0 (0) |  |  | 1 (<0.1) | 1 (<0.1) |  |
| Mesh size (cm^2^), median (IQR) | 30 (18 – 38) | 27 (18 – 36) | .680 |  | 25 (18 – 36) | 36 (25 – 42) | **<.001** |
| Minimum mesh overlap (cm), median (IQR) | 2.00 (2.00 – 3.00) | 2.00 (2.00 – 2.50) | .078 |  | 2.00 (2.00 – 2.50) | 2.00 (2.00 – 3.00) | **<.001** |
| Defect closed with suture, n (%) | 1 818 (93.2) | 411 (93.2) | .980 |  | 1 468 (93.5) | 761 (93.1) | .710 |

## Table S4: Pathology at reoperation, unadjusted rates

| **Reoperation characteristics, n (%)** | **Non-smoker N = 1 950** | **Smoker N = 441** |  | **BMI ≤ 30 kg/m^2^ N = 1 577** | **BMI > 30 kg/m^2^ N = 814** |
| --- | --- | --- | --- | --- | --- |
| Surgical site occurrence* | 38 (1.9%) | 19 (4.3%) |  | 26 (1.6%) | 31 (3.8%) |
| Surgical site infection | 31 (1.6%) | 12 (2.7%) |  | 19 (1.2%) | 24 (2.9%) |
| Superficial | 27 (1.4%) | 11 (2.5%) |  | 15 (1.0%) | 23 (2.8%) |
| Deep | 4 (0.2%) | 1 (0.2%) |  | 4 (0.3%) | 1 (0.1%) |
| Organ/space | 0 (0%) | 0 (0%) |  | 0 (0%) | 0 (0%) |
| Seroma | 1 (<0.1%) | 1 (0.2%) |  | 0 (0%) | 2 (0.2%) |
| Hematoma | 4 (0.2%) | 5 (1.1%) |  | 6 (0.4%) | 3 (0.4%) |
| Skin sinus | 7 (0.4%) | 1 (0.2%) |  | 5 (0.3%) | 3 (0.4%) |
| Suture granuloma | 1 (<0.1%) | 1 (0.2%) |  | 1 (<0.1%) | 1 (0.1%) |
| Pain | 5 (0.3%) | 1 (0.2%) |  | 5 (0.3%) | 1 (0.1%) |
| Hernia recurrence | 19 (1.0%) | 3 (0.7%) |  | 16 (1.0%) | 6 (0.7%) |
| Severe complication | 8 (0.4%) | 2 (0.5%) |  | 7 (0.4%) | 3 (0.4%) |
| Mesh infection | 6 (0.3%) | 2 (0.5%) |  | 5 (0.3%) | 3 (0.4%) |
| Bowel obstruction | 1 (<0.1%) | 0 (0%) |  | 1 (<0.1%) | 0 (0%) |
| Major bleeding | 1 (0.5%) | 0 (0%) |  | 1 (0.6%) | 0 (0%) |
| Mesh-related complications | 18 (0.9%) | 3 (0.7%) |  | 14 (0.9%) | 7 (0.9%) |

BMI: Body mass index
*Some patients were represented by more than one type of surgical site occurrence

## Figure S2: Reoperation for recurrence and non-recurrence complication after open elective mesh repair of umbilical hernia, smokers versus non-smokers

*P* values were calculated using Gray’s test

Alt text: Plot demonstrating cumulative incidences of reoperation for recurrence (A) and non-recurrence complications (B) by time after elective umbilical hernia repair with mesh. Non-recurrence complications were significantly more frequent in smokers.

Figure S3: Reoperation for recurrence and non-recurrence complication after open elective mesh repair of umbilical hernia, obese (BMI > 30 kg/m^2^) versus non-obese (≤ 30 kg/m^2^) patients

BMI: Body mass index
*P* values were calculated using Gray’s test

Alt text: Plots demonstrating cumulative incidences of reoperation for recurrence (A) and non-recurrence complications (B) by time after elective umbilical hernia repair with mesh. Non-recurrence complications were significantly more frequent in obese patients (BMI > 30 kg/m^2^).

# **Supplementary references**

1. Classification of surgical codes [Internet]. [cited 2026 Jan 26]. Available from: https://english.sundhedsdatastyrelsen.dk/health-data-and-registers/classifications
